# PrEP facilitators and barriers in substance use bridge clinics for women who engage in sex work and who use drugs

**DOI:** 10.1186/s13722-024-00476-4

**Published:** 2024-06-03

**Authors:** Miriam TH Harris, Emma Weinberger, Christine O’Brien, Mary Althoff, Samantha Paltrow-Krulwich, Jessica L. Taylor, Abigail Judge, Jeffrey H. Samet, Alexander Y. Walley, Christine M. Gunn

**Affiliations:** 1 Section of General Internal Medicine, Department of Medicine, Boston University Chobanian and Avedisian School of Medicine and Boston Medical Center, Boston, MA 02118 USA; 2 Grayken Center for Addiction, Boston Medical Center, Boston, MA 02118 USA; 3 Project Trust Boston Area Substance Abuse and Harm Reduction, Boston Medical Center, Boston, MA 02118 USA; 4 AIDS Action Committee, Cambridge, MA 02119 USA; 5 Harvard T.H. Chan School of Public Health, Boston, MA 02115 USA; 6 Department of Psychiatry, Massachusetts General Hospital, Boston, MA 02114 USA; 7 Dartmouth Institute for Health Policy and Clinical Practice, Dartmouth College, Lebanon, NH 03756 USA; 8 Department of Health Law, Policy, and Management, Boston University School of Public Health, Boston, MA 02118 USA

**Keywords:** HIV Prevention, PrEP, Women, Drug Use, Sex work

## Abstract

**Background:**

Women who engage in sex work and use drugs (WSWUD) experience disproportionate HIV risks. Substance use treatment bridge clinics offer an opportunity to increase HIV pre-exposure prophylaxis (PrEP) delivery to WSWUD, but research on best practices is lacking. Therefore, we explored facilitators and barriers to PrEP across the PrEP care continuum in these settings.

**Methods:**

Bridge clinic and affiliated harm reduction health service providers and WSWUD from Boston were recruited using passive and active outreach between December 2021 and August 2022. Participants were invited to take part in semi-structured phone or in-person interviews to explore HIV prevention and PrEP care experiences overall and within bridge clinic settings. Deductive codes were developed based on HIV risk environment frameworks and the Information-Motivation-Behavioral Skills model and inductive codes were added based on transcript review. Grounded content analysis was used to generate themes organized around the PrEP care continuum.

**Results:**

The sample included 14 providers and 25 WSWUD. Most WSWUD were aware of PrEP and more than half had initiated PrEP at some point. However, most who initiated PrEP did not report success with daily oral adherence. Providers and WSWUD described facilitators and barriers to PrEP across the steps of the care continuum: Awareness, uptake, adherence, and retention. Facilitators for WSWUD included non-stigmatizing communication with providers, rapid wraparound substance use treatment and HIV services, having a PrEP routine, and service structures to support PrEP adherence. Barriers included low HIV risk perceptions and competing drug use and survival priorities. Provider facilitators included clinical note templates prompting HIV risk assessments and training. Barriers included discomfort discussing sex work risks, competing clinical priorities, and a lack of PrEP adherence infrastructure.

**Conclusion:**

WSWUD and bridge clinic providers favored integrated HIV prevention and substance use services in harm reduction and bridge clinic settings. Harm reduction and bridge clinic programs played a key role in HIV prevention and PrEP education for WSWUD. Effective behavioral and structural interventions are still needed to improve PrEP adherence for WSWUD.

**Supplementary Information:**

The online version contains supplementary material available at 10.1186/s13722-024-00476-4.

## Background

In 2021, women accounted for 59% of the 10,572 new HIV cases attributable to either heterosexual sex or injection drug use in the United States (US) [1]. In Massachusetts, outbreaks among people who used drugs in the Northeast and Boston from 2016 to present reversed the declining HIV incidence trend from 2000 to 2014 [1–3]. Women accounted for 40% of cases in these new clusters where transmission through syringe sharing was most common (54%) followed by heterosexual and/or transactional sex transmission (46%) [2]. From 2018 to 2020, 20% of women in Massachusetts diagnosed with HIV reported injection drug use as their primary exposure, compared to 12% of men [3]. Studies show a wide range of sex work prevalence among women who use drugs, from 30 to 70% dependent on setting, but consistently demonstrate that sex work is more common among women compared to men [4, 5]. Thus, women who use drugs and engage in sex work (WSWUD) represent a particularly vulnerable group who face disproportionate HIV risks.

Structural factors, such as sex work and drug use criminalization and gendered-power imbalances, drive HIV risks among WSWUD and reduce their ability to prevent HIV through condom and/or sterile injection equipment use [6–8]. For example, among a cohort of sex workers from Baltimore, approximately 42% reported coercive condom negotiation, and 39% inconsistently used condoms [9]. Inconsistent condom use was also associated with substance use during sex work in this cohort [9]. Antiretroviral pre-exposure prophylaxis (PrEP) is a biomedical, user-controlled HIV prevention method shown to decrease sexual and injection-related HIV transmission by over 70% [10]. PrEP does not require sex or drug partner participation for its use. Along with condoms, sterile injection equipment, addiction treatment, and low-barrier HIV treatment, PrEP can be an essential HIV prevention tool for WSWUD. In 2023 the US Preventive Services Task Force (USPSTF) *Recommendation Statement* endorsed PrEP for HIV prevention and noted individuals engaged in sex work remain at particularly high-risk and should be prioritized [11]. However, US PrEP public health campaigns and clinical interventions have not prioritized women. Unsurprisingly, PrEP uptake among WSWUD remains low, estimated to be roughly 2% [12–15]. Surveillance data from Massachusetts shows that of the 10,301 individuals on PrEP in 2021, only 6.8% were women [3]. Reported barriers to PrEP for WSWUD include a lack of PrEP awareness, competing survival and drug use priorities, stigma, and a lack of access to settings where PrEP education and initiation traditionally occur [15–17].

Co-locating PrEP delivery in healthcare settings already accessed by WSWUD presents an opportunity for PrEP promotion to this high-risk but underprioritized population. Low-barrier substance use bridge clinics, herein referred to as bridge clinics, offer rapid access to substance use disorder treatment [18–21]. Bridge clinics have emerged as a model for transitional care that engages people who use drugs at risk of HIV, including WSWUD [22]. Some bridge clinics have the clinical infrastructures supportive of PrEP initiation, namely access to phlebotomy and providers with prescribing privileges [23]. Studies from a single Boston-based bridge clinic demonstrated successful PrEP initiation within this clinical setting [23, 24]. Of 204 patients who accessed this clinic between January to May 2020, 86% were assessed for injection-related HIV risks, 23% were assessed for sexual HIV risks, and 20% of eligible individuals were started on PrEP or post-exposure prophylaxis (PEP) [22, 23]. The PrEP uptake rate in the bridge clinic setting was higher than other published rates, but remains lower than desired in the setting of high local HIV transmission [13, 25]. Additionally, among the 11 people started on PrEP in this cohort, only two were women. Thus, bridge clinics offer an opportunity to increase PrEP access among WSWUD, but research on best practices to achieve that goal remains incomplete.

**Fig. 1:**
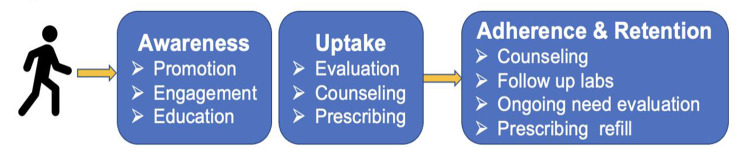
PrEP care continuum

To address this research gap, we conducted a qualitative study to broadly understand the experiences of WSWUD with PrEP overall and within bridge clinic settings. This analysis examined barriers and facilitators to PrEP access and delivery across the PrEP care continuum. The PrEP care continuum provides a framework to assess engagement or disengagement across the sequential steps of PrEP care: PrEP awareness, uptake, adherence, and retention (Fig. 1) [26, 27]. All stages can be influenced by behavioral or structural interventions that aim to increase engagement [28]. We examined experiences of both HIV prevention providers and WSWUD to identify opportunities to strengthen PrEP delivery to WSWUD at each step.

## Methods

### Aim and design

This analysis is derived from a single site qualitative study of WSWUD and health service providers, Women who Ingest drugs and engage in Sex work: Engaging in PrEP/PEP (WISE P(r)EP). The WISE P(r)EP study aimed to (1) explore experiences with PrEP among WSWUD and (2) identify potential barriers and facilitators to offering PrEP in substance use bridge clinics. The study was approved by the Boston Medical Center Institutional Review Board (H-41,804).

### Study setting

We partnered with two bridge clinics in Boston located within New England’s largest safety net hospital and largest teaching hospital respectively. Both bridge clinics offer low-barrier, rapid access to substance use treatment including medications for opioid use disorder (e.g., sublingual and injectable buprenorphine, oral and injectable naltrexone), outpatient medically managed withdrawal, referral to inpatient medically managed withdrawal facilities, harm reduction, and overdose prevention. We also partnered with a community-based harm reduction site affiliated with the safety net hospital and bridge clinic partner. The community-based program provides street-level drop-in and community-outreach to provide public health prevention services, harm reduction supplies, including syringe distribution and naloxone to clients with SUD.

### Participant recruitment

To recruit WSWUD we identified a community-based partner that serves people who use drugs and the study supported 0.20 FTE of an outreach worker’s time for active recruitment efforts. Passive and active recruitment strategies were used in the bridge clinics. Research staff attended bridge clinic meetings to introduce the study and eligibility criteria and distribute recruitment material. The outreach worker and bridge clinic staff were invited to connect WSWUD to study staff via warm hand-off or phone. Study staff then screened for eligibility and scheduled an in-person or phone interview. Eligible individuals were aged 18–65, identified as a woman, reported past year drug use, reported that they had traded sex for money, shelter, food, or drugs in the past year, and were English speaking. Individuals who reported living with HIV were excluded. The principal investigator (MTHH) recruited health service providers from the Boston-based bridge clinic partners and/or affiliated harm reduction programs through individualized email outreach. Harm reduction programs included the community-based recruitment site for WSWUD and other syringe service and homeless health outreach programs for people who use drugs that had working relationships with the bridge clinics.

### Study materials

The research team, including addiction medicine clinicians (MTHH, JT, AYW), a qualitative health services researcher (CMG), and an individual with lived expertise (MA), created a flexible interview guide (Appendix I). The interview guide used HIV risk environment frameworks to inform the exploration of HIV prevention delivery and experiences in the context of substance use and sex work. HIV risk environment theories emphasize understanding contextual factors that drive HIV risks, such as drug use or sex work policies and access to services [29, 30]. We also used the Information-Motivation-Behavioral Change (IMB) model to examine PrEP utilization facilitators and barriers. The IMB model is an intervention design framework that focuses on opportunities for behavioral change to optimize HIV prevention engagement [31, 32]. The IMB questions focused on factors related to daily oral adherence, since long-acting injectable PrEP was not meaningfully clinically available during the study timeframe. Perceptions of long-acting PrEP were systematically explored. The interview guide was evaluated by the study team and pilot tested, including identifying and removing any stigmatizing or marginalizing language. The team also developed brief questionnaires to capture demographic characteristics. Demographics collected for WSWUD included age, housing status, racial identity, gender identity, and sexual orientation. Demographics for providers included practice setting, role, gender, and racial identities. Informed consent was completed prior to study enrollment. Study staff (SD and MTHH) conducted interviews between December 2021 through August 2022 on Zoom, telephone, or in person. Interviews were audio-recorded. All participants were compensated $40 via a debit card. The mean interview length was 32 min for providers and 50 min for WSWUD.

### Analysis

Interviewer summaries were generated following interview completion to inform preliminary inductive concepts. Interviews were professionally transcribed, verified for accuracy against audio, and de-identified. The de-identified transcripts were uploaded to Nvivo 1.7.1 for data coding and analysis. The study team (MTHH, EW, MC) developed a codebook (Appendix II), containing codes pertaining to specific provider and WSWUD responses, as well as codes applicable to both interviews. Four transcripts (two provider and two WSWUD) were individually coded by three coders (MTHH, EW, and MC) to test the codebook. A second round of five transcripts (two provider and three WSWUD) were individually coded by two coders (EW and MC) to assess for agreement, with discrepancies resolved through a group consensus process [33]. The remaining 30 transcripts were then individually coded, and the team met regularly to review and resolve any coding uncertainties.

We used grounded content analysis to identify themes related to the HIV risk environment and IMB model and inductive emergent themes related to PrEP experiences in bridge clinic and community settings [34]. Next, research staff (EW and MC) created participant summaries that highlighted characteristics including PrEP knowledge, experiences, and other central topics within each interview [35]. Using these summaries and coded data, we organized participant experiences across the PrEP care continuum of awareness, uptake, adherence, and retention (Fig. 1). Throughout the analytic process codes and preliminary themes were shared with our community partners and individuals with lived experience to ensure credibility of our analysis and findings. Pseudonyms are used throughout the manuscript to protect WSWUD participant confidentiality. Additionally, age ranges (10 years) were reported to further protect WSUWD confidentiality. Providers are identified by their practice type, role, and gender only to protect their confidentiality.

## Results

In total, 39 interviews were conducted. Twenty-seven WSWUD were identified, 26 were eligible after screening, and 25 completed an interview. WSWUD participants’ characteristics were as follows (Table 1): predominately White (68%); most non-Hispanic (84%); and half unstably housed (52%). Fifteen (60%) WSWUD had care experiences at a bridge clinic. There were 15 participants (60%) who currently were taking PrEP or had done so in the past.

**Table 1:** Participant characteristics of Self-Identified women who engage in sex work and use drugs from Boston, 2021/2022 (*N* = 25)

Characteristics	*N* (%)
Age Group (Years)	
< 35 years	10 (40%)
35 + years	15 (60%)
Housing Status	
Living in a Treatment Facility	2 (8%)
Renter	10 (40%)
Staying with Someone Else	3 (12%)
Unhoused	10 (40%)
Gender Identity	
Female	24 (96%)
Trans Female/Trans Woman	1 (4%)
Hispanic/Latinx Identity	
Hispanic	4 (16%)
Not Hispanic	21 (84%)
Racial Identity^a^	
Black or African American	3 (12%)
More than One Race	2 (8%)
Other	3 (12%)
White	17 (68%)
Sexual Orientation^b^	
Heterosexual or Straight	18 (72%)
Bisexual	5 (20%)
Other	2 (8%)
Recruitment Location^c^	
Bridge Clinic Harm Reduction Organization	7 (28%) 17 (68%)
Bridge Clinic Experience	
Prior or Current Bridge Clinic Attendance	15 (60%)
PrEP Experience	
PrEP Knowledge	23 (92%)
Prior or Current PrEP/PEP Usage	15 (60%)

^a^ No participants identified as Asian, Native American/Alaska Native, or Native Hawaiian or Other Pacific Islander

^b^ No participants identified as Gay or Lesbian

^c^There was missing recruitment location data for one participant

Fifteen health service providers from Boston-based bridge clinics and/or affiliated harm reduction programs were contacted and screened;14 scheduled and completed an interview. Health service providers predominantly identified as female (64%) and White (64%). Of those interviewed, 11 (79%) had the ability to prescribe PrEP in their respective clinical settings (Table 2).

**Table 2:** Participant Characteristics of Bridge Clinic and Harm Reduction Health Service Providers from Boston, 2021/2022 (*N* = 14)

Characteristics	*N* (%)
Gender Identity^a^	
Female	9 (64%)
Male	5 (36%)
Hispanic/Latinx Identity	
Hispanic/Latinx	3 (21%)
Not Hispanic/Latinx	11 (79%)
Racial Identity^b^	
Asian	1 (7%)
Black or African American	1 (7%)
More than One Race	1 (7%)
Other	2 (14%)
White	9 (64%)
Clinical Role	
Clinician prescriber	11 (79%)
Clinician non-prescriber	2 (14%)
Harm reductionist	2 (14%)

^a^ No participants identified as transgender male or female

^b^ No participants identified as being Native American/Alaska Native or Native Hawaiian or Other Pacific Islander

Below, we describe PrEP delivery and engagement experiences across the care continuum by participant group. Table 3 summarizes the care continuum findings.

**Table 3:** PrEP Care Continuum Experiences Among Bridge Clinic and Harm Reduction Health Service Providers and Women who Engage in Sex Work and Use Drugs in Boston, 2021/2022

PrEP Care Continuum	Health Service Providers (*N* = 14)	WSWUD (*N* = 25)
Awareness		
Promotion Education/ Engagement	Many noted: 1. Public health campaigns and advertisements were not tailored to WSWUD 2. Substance use services were key to HIV prevention education for WSWUD	Most had knowledge about PrEP through social networks and substance use services, barriers included: 1. Lack of tailored messaging to WSWUD 2. Low HIV risk perceptions
	All felt PrEP education was important but noted the following competing priorities: 1. Survival 2. Substance use treatment	Incentives facilitated engagement in PrEP education
Uptake		
Evaluation/ Counseling Prescribing	All had counseling experience. 1. Note templates facilitated HIV risk assessments 2. Discomfort discussing sex work was a barrier 3. Injection drug use made phlebotomy challenging	Most had prior/current experience with PrEP/PEP: 1. Trust in care facilitated PrEP discussions 2. Stigma was a barrier to PrEP evaluations
	Providers had differing levels of comfort prescribing PrEP 1. Challenges with follow and adherence deterred initiating prescriptions	PrEP uptake was facilitated by: 1. Wrap-around substance use and HIV services 2. Same day PrEP
Adherence and Retention		
Counseling/ Follow-up Prescribing	Bridge clinic providers had less experience with PrEP adherence. Barriers to adherence across settings: 1. Lack of housing 2. Active substance use	Most WSWUD cited difficulty adhering to PrEP/PEP: 1. Competing survival priorities (drug use/safety) 2. Drug storage when unhoused For some, having a PrEP routine facilitated PrEP adherence
	Community outreach facilitated ongoing PrEP prescribing	

### PrEP awareness

Among both health service providers and WSWUD, there was a high level of awareness about PrEP: Twenty-three (92%) of women had heard of PrEP and all providers had experience with PrEP education. However, both types of participants reported that public health promotion and advertising was not inclusive of nor tailored for women, which reduced awareness:

“*[I learned about PrEP from] a doctor. He came up to me and asked me was I interested in PrEP and I’m like, “Ain’t that for gay people because of how they commercialized it. He’s like, “No. Women can take it too,” so I’m like, “Well, how do I get on it because I wanna keep myself safe?”* – Layla, 25–34 years, Multi-Racial.

*“I don’t feel like our patients really know about [PrEP]…I think we need to do a better job in educating the general population but specifically the targeted population and saying, ‘Hey this is something that’s going to impact you or you’re at risk for and there’s something we can do about it, you should ask your doctor’…I mean you’ve probably seen the ads, they don’t show anybody, ‘Hey I’m an injection drug user’…there’s stigma behind substance use and I think the message needs to change.”* – Clinician Prescriber, Male, Bridge Clinic/Primary Care.

Most women learned about PrEP through their social networks or local harm reduction, methadone, or bridge clinic services, though providers noted that their services did not necessarily advertise HIV prevention services offered. Karla (25–34 years, Other Race) described teaching a friend about PrEP based on her own PrEP experience.

*She just asked me “Why is these people taking these PrEP pills?“… I was like, “They taking this, not because they have HIV, it’s to prevent them from getting it…just so they won’t get it from the other person…” And she got interested in it because she does a lot of like tricking outside, so she got on it*.

Some providers described employing a ‘menu’ or step-wise approach to HIV prevention, which facilitated PrEP discussions:

*“[Offering] a menu makes [PrEP] more digestible… sterile injection supplies, condoms. I’m a guy that likes to have a plan, A, B, and C and so here’s the safest plan, here’s safer plan…whatever we can do in there to mitigate any kind of risk or harm would be great and it is encouraged.”* – Clinician Non-Prescriber, Male, Bridge Clinic.

*“Just because [PrEP is] high on my priority list it very well may not be high on my [patient’s] priority list, and that’s important to respect…So one tactic on an initial visit… I’ll get a little bit of their risk assessment… Then I say “Part of what we offer here, and what I recommend, is doing testing for HIV and other infections testing. Are you okay with that?”. Then we [get] the testing… and then I come back to [PrEP later].”* – Clinician Prescriber, Female, Bridge Clinic/Primary Care/Clinical Outreach.

Low HIV risk perceptions were cited as a barrier to PrEP awareness and interest by WSWUD and providers. Diana (35–44 years, Multi-Racial), had not heard about PrEP, and did not feel like she needed PrEP as she *“never had a positive [HIV] test.”* Harm reductionists and WSWUD noted that low risk perceptions could be overcome by incentivizing HIV testing as a starting point for PrEP discussion:

*“[Harm reduction program] offers a $5 Dunkin Donuts gift card to get a blood test for HIV and for other sexually transmitted diseases, which is an incentive to do it. And they’re very helpful when it comes to asking about PrEP and stuff like that, too.” – Helen, 25–34 years, White*.

Clinical priorities (e.g., treatment of substance use disorders) and women’s survival needs (e.g. being unhoused) in the setting of limited time during clinical interactions often acted as barriers to PrEP education across harm reduction and bridge clinic settings:

*“There’s definitely just time pressure. There’s a lot of people who are there to get medications for opioid use disorder. And we know that there’s a window for that, people who wait are less likely to [stay]. Part of the point of the [Bridge] clinic is to meet people right when they want treatment. And so, things like infectious disease or domestic violence I’ve shorted on.”* – Clinician Prescriber, Male, Bridge Clinic/Primary Care.

*“Why would HIV be something that [women using our services] would really care about? You know what I mean?…Some of them have diabetes, asthma, that’s untreated, a lot of other chronic conditions. And if they can’t even prioritize those that affect them on a daily basis…then it’s hard to say that they’ll be able to prioritize something that could prevent harm in the future, especially when they live their lives day-to-day are in chaos and crisis…”* – Harm Reductionist, Female, Harm Reduction Program.

In sum, while participants described overall high levels of awareness about PrEP in this study, PrEP promotion that targeted males, low HIV risk perceptions, and competing clinical and survival priorities were cited as barriers to PrEP awareness and education for this population. Strategies used to address these barriers included offering PrEP as part of a menu of prevention options and incentivizing HIV testing and risk assessments.

### Uptake

Fifteen (60%) WSWUD had experience taking PrEP at some point and all 11 of the providers who were able to prescribe PrEP had done so. Women were open to engaging in PrEP with providers in settings that were familiar and safe. Providers from harm reduction and outreach settings and WSWUD also noted the importance of empowerment during PrEP evaluations. For example, offering self-phlebotomy was way to empower patients and prevent traumatizing blood draw experiences that might have otherwise mitigated PrEP evaluations.

*“In the [harm reduction program] I liked that they were caring… They were like, “Hey, if you feel more comfortable taking your own blood…” So, they let me have the option of injecting the needle into my arm to take the blood myself, which was way easier.” –* Helen, 25–34 years, White.

*“I let my patients draw their own blood. And I don’t even know if that’s allowed, but I do. I make sure it’s in sterile settings”* – Clinician Prescriber, Female, Bridge Clinic/Primary Care/Outreach.

For providers, training and tools, such as structured HIV risk templates, facilitated PrEP eligibility assessments in a non-judgmental manner:

*“I find [the note template] very helpful, because it reminds me to do [the HIV risk assessment]…I also like it, because these questions can be difficult to talk about in a kind of non-judgmental way. And so I find if I ask them in a templated way, it feels more non-judgmental to me.”* – Clinician Prescriber, Male, Bridge Clinic/Primary Care.

Barriers to PrEP uptake cited by WSWUD included concerns about medication side effects, perceived lack of need, and mistrust in medical settings which made discussing sex work and HIV risks feel unsafe.

*“I want to know what the side effects are because right now some of them have some severe, severe, bad ones” –* Maureen, 45–54 years, White.

“*Side effects…like, I wouldn’t want to take it every day or every month If I got sick. If every time I took it, I came broke down with the flu-like symptoms or something like that…or like Pepto-Bismol commercial, like nausea, vomit, indigestion, upset stomach diarrhea. No, I don’t want any of those…” –* Tamara, 25–34 years, Black, Hispanic.

“*[With] random ER doctors I would definitely feel uncomfortable because I don’t know them. They’re there for emergency services and when they ask questions [about sex work], it makes me feel a little uncomfortable just because I don’t know if they’re going to go to the police or something.” –* Helen, 25–34 years, White.

Some male providers also cited discomfort discussing sex work risks, feeling ill equipped to address trauma that might be associated with such discussions: *“Really as MDs, we don’t know how to deal with it. We’re not trained.”* (Clinician Prescriber, Male, Bridge Clinic/Clinical Outreach).

Women in our sample with PrEP experience predominately accessed PrEP through outreach services affiliated with harm reduction programs where pills were directly dispensed to them. Providers and WSWUD both noted that programs wanting to offer PrEP needed to have ways to quickly provide medications when people were ready to initiate PrEP. For example, some bridge clinic providers noted that even though there was a pharmacy right beside the bridge clinic, not being able to dispense PrEP medications within the clinic itself created barriers to uptake for some patients. Concerns about future daily-oral PrEP adherence was also named as a barrier to prescribing, especially for individuals who lacked housing and phone access.

*“I actually feel pretty uncomfortable prescribing it, because I’m not that confident that they will adhere to [PrEP].”* – Clinician Prescriber, Male, Bridge Clinic/Primary Care.

Overall, PrEP was more readily accessed when delivered in safe, trusted clinics, and same-day medication access was provided. Concerns about side effects were barriers for patients, while some service providers noted discomfort with prescribing if they felt adherence wouldn’t follow.

### Adherence and Retention

WSWUD and health service providers described PrEP routines aligned with strong motivations to reduce HIV risks were key to daily oral adherence. For example, Imani (45–54 years, Black), who had taken PrEP for many years, described ongoing sex work as her reason for her PrEP use, noting instances of inadequate condom access or condom failure during sex work. She described pharmacy supports that facilitated her long-term PrEP adherence: *“I have a box. My medicine comes to me and so it’s already in the little thing and I just rip it. You ever seen it on TV, it’s in the box and you just rip it. The pills are already mixed for you”.* Clinicians shared similar stories where motivation and structure facilitated long-term adherence:

*“We have this one female, she’s actually stably housed at the moment but still actively using. Her partner is HIV positive. It’s unclear if he’s on ART. She’s been coming every month. [The outreach staff] are really good when she comes in about asking if she needs a [PrEP] refill or labs.”* – Clinician Prescriber, Female, Harm Reduction/Outreach.

*“I have a patient who does sex work, she very rarely injects drugs…She’s on methadone, so she’s not dealing with intense cravings or withdrawal. She’s able to plan ahead around that and she’s on PrEP. And she’s been on PrEP for years… Every three months we tend to get her to the lab or if she’s a little late, I tend not to worry so much because she’s so reliable at taking it.”* – Clinician Prescriber, Female, Bridge Clinic/Primary Care.

Both groups recognized that competing drug use and survival priorities, especially for unhoused WSWUD, hampered daily oral adherence within this population. Most participants who had PrEP experience stopped shortly after initiation due to these competing priorities.

*“Its really hard when you’re out there. You’re not thinking about [protecting yourself from HIV] you’re thinking about your next high and how you’re going to get it.”* – Isabelle, 25–34 years, White.

*“I mean, I was on the streets, I had nowhere to keep prescriptions…I don’t have safety with domestic violence when I get high. I mean if I had a safe place…. but if I’m tired, I can’t do the right thing.” –* Maureen, 45–54 years, White.

Providers noted that a lack of continuity with patients further disrupted efforts to promote PrEP adherence for WSWUD:

*“The men that we serve, we generally see them every day at the [harm reduction program]. Whereas the women…They just don’t seem to settle in the same way that the men do. And so that makes [daily observed PrEP therapy] really hard…We very much will try to be like, “Hey, if you’re expecting not to be here for a few days, let’s just give you three doses…” But the work that they do is very unpredictable and so they don’t always know that they’re going to be gone for 3–4 days.”* – Clinician Prescriber, Female, Primary care/outreach.

The bridge clinic structure, where most patients engaged with services for a short period of time and with different providers, made staying connected with such patients even more challenging as a Clinician Prescriber (Male, Bridge Clinic/Primary Care) described: “*the hardest would be when [patients] don’t have a telephone or a physical address. So there’s no like no way that I can see to get in touch with them”*. Bridge clinic providers felt they offered *“more one-off [PrEP] counseling”.*

Given the noted barriers around medication storage needed for daily oral PrEP and limited clinical contact, injectable PrEP was discussed as a potential solution to overcome some of these barriers to adherence. Though one injectable PrEP formulation was approved by the FDA at the end of 2021 [36], it was not yet clinically available in our study context, so no WSWUD or providers had personal experience but all saw the potential of an injectable form of PrEP:

*“Because you know when you’re running hard, you have no responsibility. I don’t give a flying fuck. [Long-acting PrEP] It’s a one shot, easier. You know what I mean? That would be a great thing.”* – Maureen, 45–54 years, White.

*“I think it’s just like anything that is taken chronically, I think minimizing responsibility on the patients to remember to take the medication every day. You can obviously increase adherence, increase protection just like, similar to Sublocade for treatment for opioid use disorder.”* – Clinician Prescriber, Male, Bridge Clinic/Primary Care.

Despite the benefits, participants anticipated that injectable PrEP would not fully overcome previously noted barriers including concerns about side effects and challenges with adherence:

*“I would like to try something for at least for a month or a couple of months- the pill version to make sure that it doesn’t affect me, especially my mood or my meds in any weird way…I just want to make sure that I felt fine before I would feel comfortable with getting a shot.” –* Tess, 25–30 years, White.

*“Often times where we lose people and have to restart PEP or PrEP is at one month…. So I think that would be the same if we needed follow up at the end of eight weeks [for long-acting PrEP]. I think that would really be the main obstacle.*”– Clinician Prescriber, Female, Outreach.

Despite known barriers, providers in bridge clinic settings, many of whom delivered long-acting injectable formulations of buprenorphine, felt their clinical structures would be well suited to injectable-PrEP delivery.

## Discussion

This qualitative study sought to identify barriers and facilitators across the PrEP care continuum among 25 WSWUD and 14 health service providers from harm reduction and bridge clinic settings. WSWUD and providers favored integrated HIV prevention and substance use services, as this facilitated engagement in PrEP discussions and continuing care. Despite high PrEP knowledge and experience among WSWUD, daily adherence remained challenging. Our study shed new insights to PrEP for WSWUD and for bridge clinics across the care continuum of awareness, uptake, adherence, and retention.

PrEP awareness (92%) and lifetime use (60%) among WSWUD was higher than that reported in previous literature [13, 15, 37–40]. This may reflect a combination of contextual factors and study limitations. In addition to strong public health infrastructure and longstanding insurance mandate with low uninsurance in Massachusetts, there have been local efforts in Boston to promote PrEP among harm reduction and medical providers [41]. In the neighborhood where this study was conducted PrEP was accessible at community health centers for people experiencing homelessness, multiple harm reduction programs, low barrier substance use treatment clinics, and the inpatient safety net hospital [13, 23, 25]. Furthermore, due to our recruitment strategy and topic of this qualitative study, participation may have been more appealing to WSWUD who already had PrEP awareness and experience. By comparison, only 21% of a diverse sample women sex workers from Baltimore were aware of PrEP and global estimates among women who sell sex or use drugs show awareness varying from 4 to 36% [15, 42]. However, though knowledge and access were higher in our study, both WSWUD and providers discussed a lack of inclusive PrEP messaging through commercials and public health campaigns, which remained focused on men. Consistent with other literature, women predominately learned about PrEP through their own social networks or through local substance use services [37, 43]. Thus, our findings strengthen calls for PrEP campaigns that include women and focus on both injection and sexual risks [28]. Given the importance of local substance use services in delivering HIV prevention education and care, such programs should also consider advertising their HIV prevention services more directly to WSWUD.

In terms of PrEP uptake, discussing risks associated with sex work was reported as a barrier. Women feared legal repercussions in settings where they had low trust or had experienced stigma. Male providers in particular expressed discomfort asking about sex work risks, citing lack of experience or concerns about unintentionally triggering patients’ trauma. Enhancing trauma-informed HIV risk assessment approaches among substance use providers, which have been shown to mitigate barriers to substance use and HIV prevention service engagement, may facilitate PrEP evaluations among WSWUD [44–46]. All participants favored integrated HIV prevention and substance use services as this facilitated PrEP initiation. Bridge clinics were well-positioned to fulfill this role, especially with regards to short term uptake. Note templates prompting HIV risk assessments were helpful in prompting and completing PrEP evaluations. Though bridge clinics facilitated access to phlebotomy and pharmacy services, blood draws and medication access were still noted as barriers to uptake. Phlebotomy was challenging and sometimes traumatizing for women who injected drugs. Some WSWUD and providers based in low-barrier primary care or harm reduction settings discussed the value of women drawing their own blood in facilitating PrEP evaluations. Integrating approaches that reduce trauma or facilitate self-phlebotomy as a strengths-based engagement strategy may be an important tool to increase PrEP assessments among women who inject drugs. Such tactics require further systematic study to determine their impact on PrEP engagement.

Housing instability and active drug use were cited by both providers and women as major barriers to daily oral PrEP adherence. Conversely, women who developed a routine, consistently engaged in substance use treatment, and stayed connected with harm reduction and outreach services were able to sustain PrEP adherence and follow-up care. Participants in our study portrayed that when competing drug use and survival priorities were reduced and HIV supports made available, WSWUD can and did prioritize HIV prevention and PrEP. From the provider perspective, the bridge clinic structure, where several providers might see the same patient while they await connection to longer-term care, also presented some barriers to PrEP delivery adherence support. Patient navigation, through peer or professional supports, has been shown to increase engagement in HIV and substance use treatment in other clinical settings and populations, including PrEP-specific navigation [47–52]. This is consistent with our participants’ experiences regarding the value of outreach services supporting PrEP adherence and follow-up in community settings [53]. Future research could consider assessing the integration of HIV prevention navigators or leveraging already integrated peer staff to enhance PrEP adherence and care coordination through bridge clinics.

Consistent with the literature, participants were enthusiastic about long-acting injectable-PrEP as a potential HIV prevention tool [54–56]. Both WSWUD and providers cited the possible adherence benefits of receiving PrEP injections every two months compared to daily oral PrEP. Like other marginalized populations, WSWUD noted a long-acting option would be especially beneficial in the setting of active drug use and challenges keeping medications safe while being unhoused [54, 57]. WSWUD and providers felt harm reduction programs and bridge clinics would be well-suited to delivering long-acting PrEP formulations. Experience delivering long-acting buprenorphine for opioid use disorder among substance use providers make them especially primed to provide long-acting PrEP. However, concerns about medication side effects among WSWUD and follow-up adherence among providers were noted. Leveraging existing or establishing new PrEP-outreach programs that offer ongoing engagement supports for long-acting PrEP follow-up care will likely be important for its success among WSWUD [58].

Our findings must be interpreted within the context of the study’s limitations. First, access to public health services and insurance was high in Boston compared to other urban centers or more rural areas. Second, WSWUD were recruited through harm reduction and bridge clinic programs, therefore, we may have missed women who were entirely disconnected from care and such individuals would likely have lower PrEP knowledge and experience. Our recruitment approach also impacted the racial and ethnic diversity of our study sample, which was disproportionately White. There are persistent disparities in harm reduction and SUD treatment engagement in Boston and within our recruitment settings [59, 60]. Black and Hispanic women are less likely to be connected to both harm reduction and SUD treatment thus the value of harm reduction and bridge clinic services for PrEP education and initiation reported by our participants may be overstated and/or lacking perspectives that may be unique to Black and Hispanic WSWUD [16, 61, 62]. More qualitative and quantitative research in Black and Hispanic populations are needed to better understand the impact of racial and ethnic identities on HIV prevention and PrEP to inform establishing inclusive and responsive approaches. For providers, those interviewed predominately practiced in the neighborhood in Boston that is home to a highly concentrated area of substance use and homelessness where the 2019 HIV cluster emerged. Therefore, they likely have more proficiency and interest in HIV prevention and PrEP compared to providers working in other settings. Future studies should engage substance use providers who work in diverse settings and regions to glean variable experiences with HIV prevention prioritization.

## Conclusion

Integrated HIV prevention and substance use services in trusted bridge clinic and harm reduction settings facilitated PrEP engagement among WSWUD. Training substance use providers in PrEP and integrating HIV risk assessment templates facilitating PrEP delivery are practical and useful measures to advancing such care in bridge clinic settings. Improving trauma-informed HIV risk assessments and integrating PrEP care coordination supports could further enhance PrEP uptake for WSWUD. However, even settings with supportive PrEP outreach, PrEP adherence was challenging for WSWUD who were unhoused and actively using drugs. In addition to behavioral, public health, and clinical interventions, structural interventions, such as sex work decriminalization, are also needed to reduce HIV risks and improve PrEP engagement among WSWUD.

### Electronic supplementary material

Below is the link to the electronic supplementary material.

Supplementary Material 1

Supplementary Material 2

## Data Availability

The datasets generated and/or analyzed during the current study are not publicly available due the need to protect the confidentiality of our participants but are available from the corresponding author upon request.

## Abbreviations

HIV: human immunodeficiency virus
USPSTF: US Preventive Services Task Force Recommendation Statement
PrEP: antiretroviral pre-exposure prophylaxis
PEP: antiretroviral post-exposure prophylaxis
WSWUD: women who engage in sex work and use drugs
WISE P(r)EP: Women who Ingest drugs and engage in Sex Work: Engaging in PrEP/PEP
IMB: information-motivation-behavioral change
