# Supplementary material for: PrEP facilitators and barriers in substance use bridge clinics for women who engage in sex work and who use drugs
Source: Addict Sci Clin Pract. 2024 Jun 3;19:47. doi: 10.1186/s13722-024-00476-4 (PMC11145858; doi:10.1186/s13722-024-00476-4)
Supplement: Supplementary file 1 — Supplementary Material 1 [file 13722_2024_476_MOESM1_ESM.doc]

**Interview guide: Interviews Among Key Informants**

Interview ID _____________________

Date ___________________________

*Please note: This is an interview guide, intended to be used flexibly, to allow for a conversational flow to the interview while covering the topics below. Prompts are included here as possible suggestions for elaboration if responses are short.*

**Outline**

**Introduction:**

Thank you for talking to me today. We will spend about the next 30 minutes or so talking about your thoughts about caring for women who use drugs and for women who engage in sex work in the bridge clinic (low barrier addiction clinic, like Faster Paths or the MGH Bridge Clinic). We will also explore your experiences or suggestions regarding potential barriers and facilitators to offering HIV prevention services, specifically antiviral post-exposure, and pre-exposure prophylaxis (PEP and PrEP), in bridge clinics. As a reminder, what you say here will be kept confidential. We will identify your interview by a number only and remove any names from the written version of this recording. We will also delete the recording once we have a written copy.

Do you have any questions before we begin?

| **Section 1**: **Experiences providing care to women who inject drugs and women who engage in sex work** |
| --- |


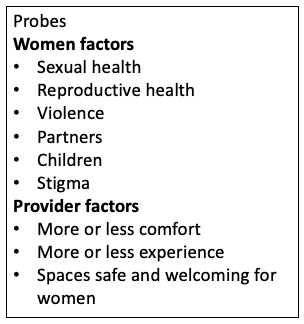


**General questions**

First, I’d like to learn about your experiences working with women who use drugs.

1. What has your experience been providing care to women who use drugs?
2. What concerns come up most often for women in your work? What do they most want help with? What services do they most request?

**Addressing Sex Work**

In prior research we’ve done, many women have disclosed that they engage in sex work (trading sex for money, drugs, safety, shelter, or other goods or services).

1. How does sex work come up with your clients, if at all? How do you address this aspect of women’s lives, if at all?
2. Sex work might feel like a difficult topic for some people (clients and/or providers) to talk about. What’s it like for you to discuss this topic?
   1. What settings are most conducive to these conversations?
   2. When might you avoid the discussion of sex work with a client?
3. What is unique about caring for women who use drugs and/or who are trading sex?

| **Section** **2**: **Current HIV prevention experiences.** |
| --- |

Next, we would like to learn more about how HIV prevention is addressed at your place of work.

1. What HIV prevention or treatment services, if any, are currently offered?
   1. To whom do you offer these services?
   2. What would prompt services to be offered to an individual in your program? (Probe for specific infection risk factors that they look for. What tools or resources do use?)
   3. How do you manage addressing HIV prevention among all the other addiction and health care needs of women accessing services?


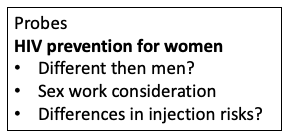


1. What is unique about HIV prevention for women? (probes)
2. What other HIV prevention services are needed for women who use drugs and women who do sex work?

| **Section 3: Facilitators/Barriers to PEP and PrEP delivery.** |
| --- |


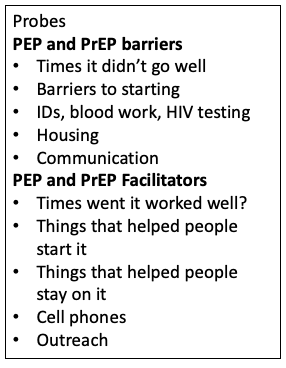
Next, we would like to learn more about your experiences with post- and pre-exposure prophylaxis (PEP and PrEP) for HIV prevention.

1. What do think about PEP and PrEP for HIV prevention?
2. What has been your experience in providing PEP and PrEP to patients/clients?
3. How confident are you providing PEP and PrEP overall?
4. Are there particular circumstances that make you more or less comfortable when providing PEP/PrEP? (probes)
5. What have you learned from your time providing PEP/PrEP that you recommend to others starting this work? (probes)
6. A once monthly or every 8-weeks injectable formulation of PrEP may be available, how do you think long acting injectable PrEP would work for women who use drugs and do sex work?

| **Section 4: Information, motivation, behavioral skills needed for PEP and PrEP services.** |
| --- |

**Identification and eligibility**

1. For whom should HIV prevention and PEP and PrEP be offered?
2. What has or would help you identify people eligible for PEP or PrEP?


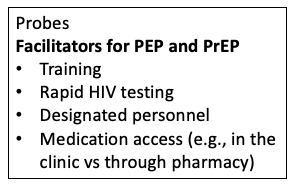
**Counseling and delivery**

1. What would help you to provide PEP and PrEP? (probes)
2. Who [in your clinic?] do you think should provide PEP and PrEP?
3. What tools do you think your place of work would need to effectively offer PEP and PrEP? (probes)

| **Conclusion** |
| --- |

Thank you for sharing your thoughts about HIV prevention and PEP/PrEP care for women. Is there anything else that we have not addressed today that you would like to share?

If we could write down one thing from today that you would want us to remember about helping women access HIV prevention services?

**Interview guide: Interviews among Women who Use Drugs & Engage in Sex Work**

Interview ID _____________________

Date ___________________________

*Please note: This is an interview guide, intended to be used flexibly, to allow for a conversational flow of the interview while covering the topics below. Prompts are included here as possible suggestions for elaboration if responses are short. The interview is designed to be driven by the participants.*

**Outline**

**Introduction:**

Thank you for talking to me today. We will spend about the next 45 minutes or so talking about your experiences being a woman using drugs. We will ask about how programs can make it easier for women to access support services for drug use, HIV prevention, and other services that might be of interest to you. As a reminder, what you say here will be kept confidential. We will identify your interview by a number only and remove any names from the written version of this recording. We will also delete the recording once we have a written copy.

What questions do you have before we begin?

**Outline**:

| **Section 1 : Social structural context** |
| --- |

**General questions**

First, I’d like to learn about you.

1. Tell me a bit about being a woman who uses drugs.
2. What do you worry most about being a woman who uses drugs?

**Sex work**

If its ok, we would like to learn more about your experiences trading sex for things like money, shelter, safety, or drugs. People call this different thing, e.g. sex work, going on dates, working the street.

1. What do you call this and what language do you want me to use today in our conversation?
2. What do you worry most about in terms of your health and/or safety when doing (sex work or other phrase)
3. How do you keep yourself safe when doing (sex work or other phrase)
4. Do you mind sharing how doing (sex work or other phrase) relates to your drug use, if at all?
5. What services, if any, help you stay safe?
6. What other services would be most helpful?
7. How would you like to be asked about sex risks like from doing (sex work or other phrase)?

**Drug use**

Thank you for sharing with me. Now I would like to learn about your experiences using drugs.

1. What do you worry most about in terms of your health or safety when using drugs?
   1. How do you usually use drugs (e.g. injection, smoking, or sniffing)?
   2. How do you keep yourself safe when using drugs?
2. How would you like to be asked by a healthcare provider or outreach worker about using drugs?
   1. What have your experiences been like talking to a healthcare provider (e.g. doctor, nurse etc.) or outreach worker about using drugs?
      1. What went well in that appointment?
      2. What could have been better in that appointment?

**HIV risks**

I’d like to learn more a bit more about your specific health and safety concerns about HIV.

1.
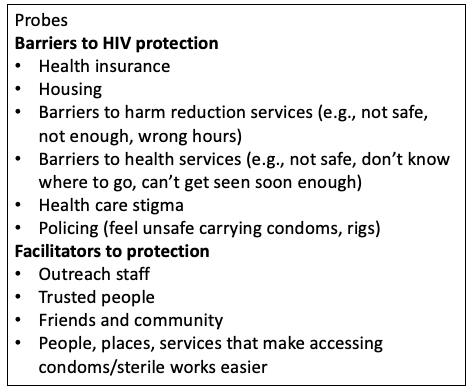
How worried are you about HIV? How do you deal or think about this?
2. How do you protect yourself from HIV?
3. What makes it hard to protect yourself from HIV? (probes)
4. What has made it easier to protect yourself from HIV? (probes)
5. Tell me about a time someone asked you about your HIV risks (e.g., outreach worker or doctor).
   1. Who was it?
   2. What was good about this?
   3. What could have been done better?
   4. In the future, who would you be most comfortable talking to about HIV risks? Why?
6. What do you think about including HIV services (e.g. testing, condoms, works) with addiction treatment (e.g. methadone, buprenorphine, detox)?

| **Section 2: Information motivation behavioral (IMB) skills needed for PEP and PrEP uptake and adherence** |
| --- |

**PrEP and PEP Knowledge**


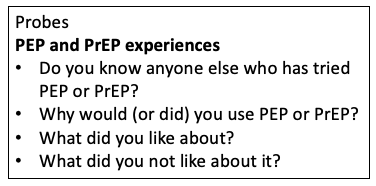
I would like to learn more about your thoughts about HIV protection.

**PEP and PrEP experiences**

1. Have you had any experiences with PEP or PrEP (or antiviral post-exposure and pre-exposure prophylaxis medications for preventing HIV)? (probes)
2. **If not,** what have you heard, if anything, about PEP or PrEP?
   1. ***If they have not heard about PEP/PrEP***: Would it be okay if I told you a little bit about PEP or PrEP?
      1. [*PEP or PrEP are medications that can help protect you from HIV. Post-exposure prophylaxis or PEP is used after an exposure happens – for example, after having sex without a condom or sharing works or needles with someone who has HIV or might have HIV. PEP is prescribed by a doctor or other health professional and should be started as soon as possible after an exposure, ideally within three days. PEP involves taking a pill (or pills) every day for a month. Pre-exposure prophylaxis or PrEP, on the other hand is for people who may have HIV exposures in the future. PrEP can help people avoid HIV infection if they have sex with men without condoms, if they share works or syringes, or both. PrEP involves taking 1 pill per day, and people continue it until they stop having any possible HIV exposures for 6 months [aren’t having condomless sex or sharing works/injection equipment].*
   2. ***If they have heard about PEP/PrEP***: where did you first learn about PEP and PrEP?
      1. Who has talked to you about PEP/PrEP since you first heard about them?
3. **(For everyone)** Who is the best person to share information about PEP or PrEP? (e.g., peer worker/recovery coach, doctor, nurse, syringe service worker)
4. In your view, how helpful are these medications at preventing HIV?
5. What would you want to know or did you want to know about these medicines before taking them?
   1. What would help you to take these medications?
6. What questions do you still have about PEP/PrEP?

**Facilitators and Barriers to PEP and PrEP (ask everyone)**


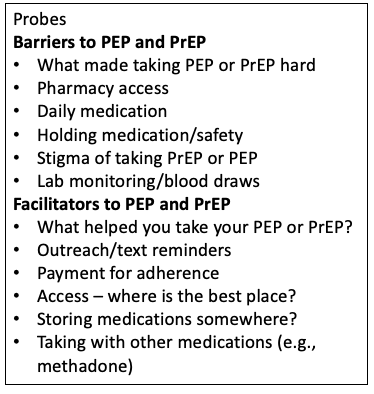


1. Do you think PEP and PrEP are good ways to protect yourself from HIV? Why or why not? (probes)
2. What would help you to take PEP and PrEP? (probes)
   1. What else would be supportive in helping you take PEP or PrEP?
3. What would motivate you to take PEP and PrEP? (probes)
4. In the future, PrEP may be available **as an injection you take every other month** (every 8 weeks)?
   1. What might be better about an injection for HIV protection compared to taking a daily pill?
   2. What might harder about an injection for HIV protection compared to taking a daily pill?
   3. Where would you want to go to get a PrEP injection? Why?
   4. What would help you get an injection every other month?

| **Section 3: Experiences with and Preferences for PEP and PrEP delivery in Bridge clinics** |
| --- |

Next, I would like to switch gears a bit and learn more about your experiences with bridge clinics. *Bridge clinics are walk in addiction treatment clinics that provide medication treatment for addiction, like suboxone, and other harm reduction services. Some clinics in Boston you may have heard of include Faster Paths, MGH Bridge clinic, or Bridge over troubled water van.*

1. Have you ever been in a clinic like this?
   1.
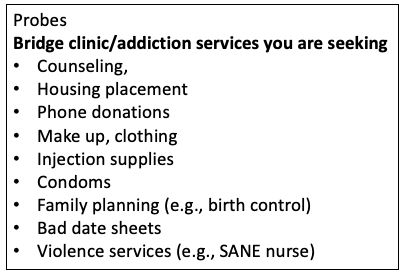
**If yes**, tell me a little about those experiences (e.g., What brought you to the clinic? What did you like about getting care there? What was hard about getting care there?)
      1. What services did you get from the clinic?
      2. What services do you wish these clinics offered, or how could they better address your needs? (probes)
   2. **If not**, why?
2. What do you think about getting HIV prevention care at a bridge clinic?
3. How could bridge clinics better meet the needs of women who use drugs or women who are trading sex? (probes)

| **Conclusion** |
| --- |

Thank you for chatting with me today about your experiences! It was great to hear your perspective.

If there is one thing we should remember from today’s conversation, what would it be?

Is there anything else that we have not addressed today that you would like to share? I appreciate you sharing your time and thoughts with me today.
