# Supplementary material for: PrEP facilitators and barriers in substance use bridge clinics for women who engage in sex work and who use drugs
Source: Addict Sci Clin Pract. 2024 Jun 3;19:47. doi: 10.1186/s13722-024-00476-4 (PMC11145858; doi:10.1186/s13722-024-00476-4)
Supplement: Supplementary file 2 — Supplementary Material 2 [file 13722_2024_476_MOESM2_ESM.docx]

**Codebook -WISE PrEP**

| Name | Description |
| --- | --- |
| 01 Sex work | Description of sex work, including the language used to describe exchanging sex and its relationship to substance use (parent node-do not code here). |
| 01a Sex work participation | Description: Any references to participation in sex work. Inclusion: descriptions of women’s participation in sex work, perception of participation in sex work, or identity as a sex worker. Exclusion: Descriptions of stigma or discrimination experienced related to sex work (code 04). |
| 01b Relationship of sex work and substance use | Description: References to how substance use impacts sex work and vice versa as well as references to a lack of relationship between the two. Inclusion: Descriptions of doing sex work to support substance use. Exclusion: Descriptions discrimination/stigma related to sex work or drug use (code 04). |
| 02 Safety while using drugs and doing sex work | References to experiences of violence and safety strategies SWWIDs use to protect themselves (parent node-do not code here). |
| 02a Experiences of physical and sexual violence | Description: References to experiences of physical/sexual violence or intimidation. Inclusion: descriptions of instances of assault, rape, coercion, or verbal abuse. Exclusion: descriptions of police or state violence (code 03) or strategies used to keep safe (code 02b). |
| 02b Safety strategies | Description: References to strategies (or lack thereof) undertaken by women to keep themselves safe. Inclusion: descriptions of behaviors or systems used to increase safety, for example using drugs with a trusted friend or using a buddy system while doing dates. Include descriptions of strategies women have found unhelpful. Include aspects related to intuition or gut feeling. Include references to not using safety strategies. Exclusion: descriptions of emotional/psychological safety provided by SWWIDs’ social support networks (code 07b). Descriptions of safety strategies specifically related to sexual health safety or HIV risk prevention strategies (e.g. condom use) (code 02c) |
| 02c Sexual Health safety strategies and HIV Risk prevention | Description: references to strategies (or lack thereof) undertaken by women to safeguard their sexual health or reduce their HIV risk while participating in sex work or using drugs. Inclusion: descriptions of behaviors or systems used increase sexual health safety or HIV risk safety (e.g. using clean needles or insisting on condom use during intercourse). Include lack of safety strategies (e.g. having unprotected sex with a partner who has HIV). Exclusion: Descriptions of safety strategies not related to sexual health or HIV risk prevention (02b) |
| 03 Policing and criminalization | Description: References to positive or negative policing experiences, the criminal justice system, and other state regulatory agencies (e.g. DCF). Inclusion: descriptions of police surveillance, violence, or other system/state actor experiences (e.g. security guards). Include the impact these have (positive or negative) on SWWID’s and HIV prevention (e.g. not wanting to go to a needle exchange program after hearing there was an ICE raid nearby). Exclusion: Descriptions relating specifically to stigma/discrimination (code 04). |
| 04 Stigma/discrimination experienced by SWWIDs | Description: references to experiences of stigmatization/discrimination among SWWIDs. Inclusion: any descriptions of stigmatization/discrimination from social networks, health care systems, or other personal connections. Include discrimination related to race, gender, and other intersecting identities. Exclusion: Police/system specific violence/discrimination (code 03). |
| 05 Strength and resilience of SWWIDs | Description: any reference to the strength or resilience of SWWIDs. Inclusion: any description made by SWWIDs or providers regarding the resilience of SWWIDs and sense of empowerment derived from being a women/sex worker. Exclusion: descriptions of SWWID’s social support system (code 07b) |
| 06 Service Provider and SWWID Communication | References to communication facilitators or barriers between service providers and SWWIDs(parent node-do not code here). |
| 06a General Communication Strategies | Descriptions: general references to communication experiences between clients and service providers. Inclusion: descriptions of what settings, personnel, or experiences facilitated communication between service providers and SWWIDs in general conversations. Include descriptions where the conversation’s context is not explicit. Exclusion: communication strategies related to sex work (06b) or drug use (06d) and communication related to sex work terminology (06c) |
| 06b Client-Provider communication about sex work | Description: references to communication experiences between clients and service provider regarding sex work. Inclusion: descriptions of what settings, personnel, or experiences facilitated or impeded discussing sex work and sexual risks. Exclusion: descriptions of SWWID’s preferred sex work terminology (code 06c) and general communication strategies (06a). |
| 06c Preferred sex work terminology | Description: References to preferred sex work terminology Inclusion: Descriptions of how women describe sex work and their preferred language to discuss sex work and their sexual risks with providers (harm reduction/med/etc.). Exclusion: Descriptions of communication around sex work not explicitly regarding terminology (code 06b) |
| 06d Client-Provider communication about drug use | Description: References communication experiences between clients and providers drug use and injection risks. Inclusion: descriptions of what settings, personnel, or experiences facilitated or impeded discussing drug use/drug use risks. Exclusion: general communication strategies (06a) |
| 07 Social structural facilitators and barriers to services | References to socio-structural barriers and facilitators that impact engagement with services and/or health priorities (parent node—do not code here). |
| 07a Housing and other social determinants of health | Description: references to the impact housing/shelter and other social determinants have, such as lack of transportation or health insurance, on service access and engagement with HIV prevention. Inclusion: descriptions of challenges keeping medications safe while in a shelter or descriptions how getting housing made accessing service/prioritizing HIV prevention easier. Exclusion: descriptions of housing in general and lack of housing as a reason to use drugs/do sex work (code 01a or 01b). descriptions related to surviving day-to-day and how that impacts SWWID’s ability to engage in care or other services (07c) |
| 07b Social network of SWWIDs | Description: any reference to the social networks of SWWIDs including other SWWIDs, family members, romantic partners, friends, neighbors, etc. and their impact on engagement with health or HIV prevention priorities Inclusion: descriptions of any benefits/barriers SWWIDs’ social networks provide in access to services or health prioritization. Exclusion: any description of social supports as a safety strategies used to prevent physical or sexual violence (code 02b). |
| 07c Means of Survival/Competing Priorities | Description: any reference to SWWID’s difficulty of survival or the struggles of living “day to day” as competing priorities to their health. Inclusion: descriptions that comment on how SWWIDs’ priorities are focused on survival or getting to the next day. Include how this impacts their ability to engage in health care or other services. Exclusion: explicit references to housing or other social determinants of health (07a) |
| 08 Health Service Structures and SWWID engagement | References to the “positives and negatives” of various health service structures as they relate to SWWID engagement and access (parent node—do not code here) |
| 08a General health service structures and SWWID Engagement | Description: references to structural aspects of service delivery as they relate to SWWID engagement/access. Inclusion: descriptions of SWWIDs’ experiences with service location, personnel, hours, etc. Example: a SWWID saying “I like the staff at this program” or “they have tea and coffee and let you just hang out”. Exclusion: any KIs’ experience providing HIV prevention services at bridge clinic specifically (code 09a). Descriptions of locations specifically providing HIV care (08b) or Bridge Clinic care (08c). Descriptions to current health or social services SWWIDs are currently using (08d) |
| 08b HIV service structures and SWWID engagement | Description: references to structural aspects of HIV service delivery as they relate to SWWID engagement/access. Inclusion: descriptions of SWWIDs’ experiences with HIV service location, personnel, hours, etc. Exclusion: any KIs experience providing HIV prevention services at bridge clinic specifically (code 09b). General descriptions of health service structures and their impact on care (08a), descriptions of HIV service care at a Bridge Clinic (08c), or descriptions to current health or social services SWWIDs are currently using (08d) |
| 08c Bridge Clinic structure and SWWID engagement | Description: references to structural aspects of service delivery at a Bridge Clinic as they relate to SWWID access/engagement. Inclusion: descriptions SWWIDs’ experiences regarding Bridge Clinic location, personnel, hours, etc. Exclusion: any KIs’ experience providing services at a Bridge Clinic (code 09a). general descriptions of health service structures and their impact on care (08a), descriptions of HIV service care outside of a Bridge Clinic (08b), or descriptions to current health or social services SWWIDs are currently using (08d) |
| 08d Services that SWWIDs are currently utilizing | Description: references to services that SWWIDs are currently using . Inclusion: concrete descriptions about services being used by SWWIDs and aspects that the SWWID feels help with engagement (or lack thereof). Exclusion: descriptions about services sought or not sought by SWWIDs (10a and 10b) |
| 09 HIV prevention service delivery (KI codes only) | Bridge clinics and HIV prevention services (parent node—do not code here). |
| 09a Bridge clinic care | Description: KIs’ experiences providing care in bridge clinics. Inclusion: any descriptions by KIs about bridge clinics. Exclusion: Descriptions regarding facilitators or barriers (code 09c) to offering PrEP/PEP care at bridge clinics. Descriptions regarding providing general HIV prevention care at bridge clinics (09b). Descriptions regarding SWWIDs’ experiences at bridge clinics (08c). |
| 09b HIV Preventative Care at Bridge Clinics | Descriptions: KIs’ experiences providing HIV prevention care in bridge clinics. Inclusion: any description by KIs about providing HIV prevention care in a bridge clinic service setting, both facilitators and barriers. Exclusion: descriptions specifically regarding PrEP/PEP delivery facilitators/barriers (09c). general descriptions of bridge clinic care (09a) |
| 09c Clinical/service facilitators and barriers to PrEP/PEP delivery | Description: KIs experiences or perceived facilitators of and barriers to providing PrEP/PEP care. Inclusion: descriptions from KIs including any structural, societal, provider knowledge/comfort, or programmatic facilitators for providing PrEP/PEP care. descriptions from KIs including any structural, societal, or programmatic barriers for providing PrEP/PEP care in bridge clinics. Exclusion: any descriptions regarding general experiences with bridge clinics (code 09a), descriptions of perceived SWWID motivations for PrEP/PEP usage (code 12) or perceived SWWID perceptions of PrEP/PEP (code 11b). |
| 10 Services SWWIDs Seek | References to desired or undesired services for SWWIDs. References to services women or KI seek but do not have actual experiences with (parent node—do not code here). |
| 10a General services SWWIDs seek | Description: references to general services sought or not sought by SWWIDs. Inclusion: descriptions of proposed services SWWIDs are seeking, such as mental health care or primary care access, more outreach etc. Include services that SWWIDs do not seek (e.g. I don’t want to attend a therapy group). Exclusion: descriptions that specifically speak to services that are desired regarding PrEP/PEP (code 10b) or references to lived service experiences. Descriptions of services SWWIDs are currently utilizing (08d) |
| 10b PrEP/PEP services SWWIDs seek | Description: references to PrEP/PEP specific services sought or not sought for SWWIDs. Inclusion: descriptions by KIs or SWWIDs regarding proposed services that would help PrEP/PEP engagement. Descriptions regarding proposed services for PrEP/PEP engagement that SWWIDs do not want. Exclusion: descriptions that specifically speak to desiring general medical services (code 10a) or references to lived PrEP/PEP service experiences. |
| 11 PrEP/PEP knowledge and perceptions | Descriptions of PrEP/PEP as a HIV prevention tool (parent node—do not code here). |
| 11a Information on PrEP/PEP | Description: references to information that SWWID have on PrEP/PEP. Inclusion: descriptions from SWWID or KI regarding PrEP/PEP for HIV prevention for SWWID. Include references on information regarding SWWID knowledge on where access PrEP/Pep, the use of PrEP/PEP, side effects, etc. Include descriptions about not being provided with information (e.g. “I have never heard of PrEP”) and KI comments about SWWID lack of awareness of PrEP/PEP. Exclusion: any descriptions regarding perceptions held by the SWWID regarding PrEP/PEP (code 11b), or KI descriptions of PEP/PrEP facilitators (code 09b) or barriers (code 09c). |
| 11b Perceptions of PrEP/PEP | Description: perceptions of PrEP/PEP as an HIV prevention tool. Inclusion: descriptions from SWWIDs and KI regarding their perceptions of PrEP/PEP medication (e.g. “I thought PrEP was only for gay men” or “I think it would be good because it protects during sex and injection”) Exclusion: descriptions of information about PrEP/PEP that the SWWID has received (code 11a). |
| 12 Motivation for PrEP and PEP usage | Description: references to motivation in SWWIDs for PrEP/PEP usage. Inclusion: any description by KI or SWWIDs of internal, individual, social, or community motivation for PrEP/PEP usage (e.g. “my doctor says I should take PrEP, so I’ll try it out”). Include descriptions of aspects that reduce motivation. Include descriptions of rewards (e.g. free coffee) as motivating factors. Exclusion: descriptions of motivation for general medical care services. |
| 13 Behavioral skills needed for PrEP/PEP usage | Description: References to behaviors that either promote or deter PrEP/PEP usage in SWWIDs. Inclusion: any description of a behavior that either promotes or deters usage of PrEP/PEP in SWWID populations (e.g. setting a calendar reminder on their phone to take PrEP daily). Exclusion: descriptions related to SWWID knowledge (11a) or perception (11b) of PrEP/PEP. |
| 14 Long-Acting Injectable PrEP (LA-PrEP) | Description: any reference made to LA-PrEP. Inclusion: descriptions regarding benefits/drawbacks/general comments related to LA-PrEP. Exclusion: any descriptions about regular PrEP, not long-acting injectable PrEP. |
